# Breeding and characterization of a new sweetpotato cultivar, ‘Miyaakari’, with bright yellow flesh for confectionery processing

**DOI:** 10.1270/jsbbs.25017

**Published:** 2025-10-18

**Authors:** Keisuke Suematsu, Yumi Kai, Rie Kurata, Akira Kobayashi, Takeo Sakaigaichi, Yukari Kawata, Tetsufumi Sakai, Yasuhiro Takahata, Masaru Yoshinaga, Kenji Katayama, Toshiro Fujita

**Affiliations:** 1 Kyushu Okinawa Agricultural Research Center, National Agriculture and Food Research Organization, Yokoichi 6651-2 Miyakonojo, Miyazaki 885-0091, Japan

**Keywords:** Miyaakari, sweetpotato, confectionery processing, flesh color, carotenoid

## Abstract

We developed a new sweetpotato cultivar, ‘Miyaakari’, for confectionery processing. It was released in 2023. Herein we observed that Miyaakari shows a significantly higher yield compared to two major sweetpotato cultivars in Japan, ‘Kokei No. 14’ and ‘Beniharuka’. Miyaakari has a higher carotenoid content than Kokei No. 14 and Beniharuka, and the flesh color of heat-cooked Miyaakari sweetpotato is bright yellow. The quality of heat-cooked Miyaakari sweetpotato (i.e., hardness and sweetness) is more stable during storage compared to Beniharuka. These characteristics are suitable for food processing. Indeed, a primary food manufacturer concluded that Miyaakari is suitable for ingredients of paste and diced food products. Miyaakari will thus both contribute to an increased production of ingredients for confectionery processing and improve the quality of sweetpotato sweets.

## Introduction

Sweetpotato (*Ipomoea batatas*) is used for various processed foods. Sweets or snacks made of sweetpotato are popular in Japan. In 2022, the production of sweetpotato for processed foods was 92,500 tons, and this is the third highest amount following those for table use and alcohol (MAFF 2024). Most of the sweetpotato for processed foods was used for confectioneries in Japan’s Kyushu region and for steamed and dried sweetpotato slices (*hoshi-imo*) in the Kanto region. Sweetpotato for confectionery undergoes primary processing to paste or a diced form, and it is then sold as ingredients of various sweets such as *yokan* (Japanese jelly-like sweets) and *manju* (Japanese bun).

The most important characteristics of sweetpotato cultivars for confectionery processing are (1) high yield, (2) bright and strong yellow-flesh color, and (3) minimal change in quality during storage. Sweetpotato for processing tend to be sold at a lower price than that for table use (MAFF 2024), and a higher yield per unit area is thus required for the sustainable production by farmers of sweetpotatoes for processed foods. Regarding the quality of processed foods’ ingredients, brighter and stronger yellow-flesh color has been preferred by confectionery manufacturers because the appearance of the confectioneries is valued (Takano and Ino 2012). The stability of sweetpotatoes’ flesh quality during storage is also required by confectionery manufacturers. The post-harvest storage of sweetpotatoes alters the properties of heat-cooked sweetpotatoes. After storing raw sweetpotatoes, the hardness and sweetness of heat-cooked sweetpotatoes change with significant differences depending on the cultivar (Ando *et al.* 2018, Shimozono *et al.* 1994). Cultivars with flesh that is susceptible to softening by storage are not suitable for processing because the softened status reduces the shape retention of confectionery products (Takano and Ino 2012).

‘Kokei No. 14’ is one of the most popular sweetpotato cultivars for confectionery processing in Japan because the texture of its heat-cooked sweetpotato is relatively unaffected by storage and its taste and flavor are preferred by consumers. However, the yield level of Kokei No. 14, which was released in 1945, is lower than that of sweetpotato cultivars released later such as ‘Benimasari’ and ‘Beniharuka’ (Katayama *et al.* 2017). Kokei No. 14 also has some demerits; for example, it is susceptible to the root-knot nematode, and it is easy to deform the shape of its storage roots. Beniharuka is a popular cultivar in Japan for table use (Kai *et al.* 2010). The production of Beniharuka in Japan increased recently and became the highest among all sweetpotato cultivars cultivated in Japan in 2023 (MAFF 2024). Stored storage root of Beniharuka is greatly softened by heat-cooking (Ando *et al.* 2018), and the flesh color of Beniharuka is cream (Ishiguro *et al.* 2010). Although Beniharuka has a high yield level, it is not suitable for paste or diced use in ingredients. New sweetpotato cultivars with high yields that are suitable for confectionery processing have thus been desired as alternatives to Kokei No. 14 and Beniharuka in Japan.

Here, we developed a new sweetpotato cultivar, ‘Miyaakari’, the yield of which is higher than that of Kokei No. 14. The flesh color of heat-cooked Miyaakari is bright and strong yellow compared to those of Kokei No. 14 and Beniharuka. Thus, Miyaakari is expected to be widely used as an ingredient in both Japanese-style and Western-style sweets.

## Materials and Methods

### Breeding procedures

**Fig. 1.**
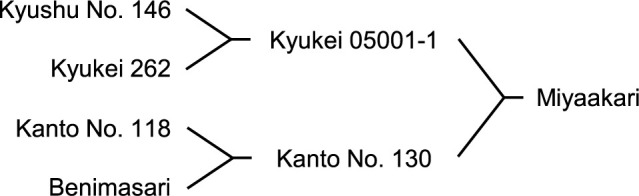
The pedigree of Miyaakari.

Fig. 1 depicts the pedigree of Miyaakari. The female parent Kyukei 05001-1 tastes good, and its flesh color tends not to be discolored in post-cooking. The male parent Kanto No. 130 has a good appearance with a deep red-purple skin color.

Total 772 seeds were obtained in 2010 by artificial pollination between Kyukei 05001-1 and Kanto No. 130. All the seeds were sown in a greenhouse, and then 164 stem cuttings from the germinated seedlings were transplanted to a field at Kyushu Okinawa Agricultural Research Center, NARO, Miyakonojo, Miyazaki, Japan (31°45 N, 131°00 E) and grown in 2011. Based on the size, shape, and number of storage roots, 17 clones were visually selected at harvest. The selected clones were propagated from a storage root and further tested for preliminary trials over a 3-year period (2012–2014) for the evaluations of the cultivar’s yield potential and the appearance and taste of the storage roots. One clone was selected based on the preliminary trials and named ‘Kyukei 333’. A yield trial of Kyukei 333 was conducted in 2015, and the local adaptability of Kyukei 333 was evaluated at four locations in Japan: Tokushima, Miyazaki, Kagoshima, and Okinawa prefectures.

Based on the evaluation results, Kyukei 333 was renamed ‘Kyushu No. 186’, and it underwent further 7-year yield trials (2016–2022). The cultivation conditions of each year are presented in Supplemental Table 1. The suitability of heat-cooked sweetpotato for processing paste and dice was evaluated for five years (2016–2020) by Agriprocess Miyazaki Company, Limited (Miyazaki, Japan), a member of research group for processing quality of sweetpotato supported by the Japan Root and Tuber Crops Development Association Inc. Foundation (Tokyo). Kyushu No. 186 was applied for Japanese variety registration as Miyaakari in 2023.

### Evaluations of color, carotenoid content, and composition

The colors of steamed sweetpotato of the three cultivars Miyaakari, Kokei No. 14, and Beniharuka were evaluated in 2018, 2019, and 2020. Storage roots sampled from each of three plots of each cultivar were steamed for approx. 50 min, and paste was made of the steamed sweetpotato by straining using a 1-mm mesh strainer. The L*a*b* color values of the pastes were measured by a spectrophotometer (CM-2600d, Konica Minolta, Tokyo).

The total carotenoid content of Miyaakari, Kokei No. 14, and Beniharuka was evaluated in 2018, 2019, and 2022 as described by Ishiguro *et al.* (2010). Raw storage roots sampled from each of three plots of each cultivar were frozen and dried. One gram of the freeze-dry powder was added with 3 mL acetone and mixed well. The mixture was centrifuged at 1500 g for 10 min, and 1 mL of supernatant was taken. Once again, the rest was added with 3 mL acetone and mixed well, and the mixture was centrifuged at 1500 g for 10 min. Three mL of supernatant was taken and combined with the previous 1-mL extract. The absorbance of the extract at 360–500 nm was measured by a spectrophotometer (UV-1700, Shimadzu, Kyoto, Japan). The total carotenoid content was estimated from the absorbance at λ max of the extract using the extinction coefficient of *E* = 2500.

The extracts mentioned above were also used to assess the carotenoid compositions of Miyaakari, Kokei No.14 and Beniharuka according to Ishiguro *et al.* (2010). The extract was dried by N_2_ gas, and the residue was dissolved by 1 mL of tetrahydrofuran containing 0.1% butylated hydroxytoluene and then filtered through a 0.2-μm membrane filter. The carotenoid compositions contained in the solution were separated and detected by a high-performance liquid chromatography (HPLC) system that consisted of a DUG-14A degasser, a SIL-10A XL auto-injector, a CTO-10AC column oven, an SPD-M10AVP diode array detector, a CBM-20A communications bus module, and two LC-10AT pumps (Shimadzu, Kyoto, Japan). The system was controlled with the use of a Lab Solution ver. 5.73 workstation (Shimadzu). A reversed-phase column was used (Wakopack Navi C30-5, 250 × 3.0 mm i.d., 5 μm, Wako Chemicals, Osaka, Japan). The carotenoid composition of each peak was determined based on the results reported by Ishiguro *et al.* (2010).

### Soluble sugar contents and the texture of the steamed sweetpotato

In 2018–2020, the soluble sugar contents of steamed Miyaakari, Kokei No. 14, and Beniharuka sweetpotato were evaluated using storage roots stored for 1 week and 12 weeks under dark and naturally cool conditions in a storage house at approximately 18°C. Storage roots sampled from each of three plots of each cultivar were steamed for approx. 50 min and then frozen and dried. Subsequently, 5 mL of 80% ethanol was added to 250 mg of the freeze-dried powder and mixed well. The mixtures were heated by boiling water for 5 min and then left for 2 hr at room temperature. The supernatant was taken and filtered through a 0.2-μm membrane filter. Sugars in the filtrates were separated and detected by an HPLC system consisting of a DUG-12A degasser, a SIL-10A XL auto-injector, a CTO-10A column oven, a RID-M10A refractive index detector, a SCL-10AVP system controller, and two LC-10AD pumps (Shimadzu). The system was controlled using the Lab Solution ver. 5.73 workstation. A hydrophilic interaction chromatography (HILIC) column was used (Shodex Asahipak NH2P-50 4E, 250 × 4.6 mm i.d., 5 μm, Resonac Holdings Co., Tokyo). The sugar composition and content were determined based on standards including glucose, fructose, sucrose, and maltose. Based on the relative sweetness of each sugar composition at 40°C (Ando *et al.* 2018), the sweetness index was calculated by following formula:

0.55*glucose (g/100 g FW [fresh weight])  + 1.00*fructose (g/100 g FW)  + 1.00*sucrose (g/100 g FW)  + 0.35*maltose (g/100 g FW)

The texture of Miyaakari and Beniharuka steamed sweetpotato was evaluated in 2018 using storage roots stored for 2 days and 18 weeks. Five storage roots for each of three replicates were steamed for 1 hr. After cooling at room temperature, two 2-cm cross-sections were cut out of each storage root, and the cross-sections were further cut into 2-cm blocks. The maximum stress when the block was cut from the top to 1.6-cm depth using a V-shape plunger was measured by a creep meter (Rheoner II, Yamaden, Tokyo).

### Statistical analysis

We performed a two-way analysis of variance (ANOVA) for agronomic traits (the total yield, the average size of a storage root, the number of storage roots per hill, and the dry matter content) among the three cultivars and among the years and for the sugar content and texture among the cultivars and among the storage periods. A one-way ANOVA was performed for the analysis of the color and carotenoid content among the cultivars. Tukey’s HSD test was used to identify significant differences among the cultivars at *p* < 0.05. All analyses were performed in R ver. 4.1.2.

## Results

### Morphological description

**Fig. 2.**
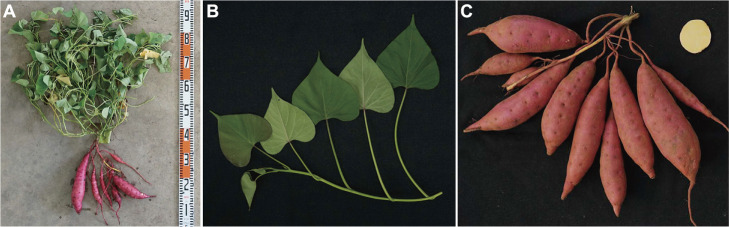
Photographs of the whole plant on 24 Aug 2022 (A), leaf on 29 Jun 2022 (B), and storage roots on 30 Sep 2022 (C) of Miyaakari. All the photographs were taken at Kyushu Okinawa Agricultural Research Center, NARO, Miyakonojo, Miyazaki.

We evaluated the cultivars’ morphological characteristics of Miyaakari according to the test guidelines of The International Union for the Protection of New Varieties of Plants (UPOV 2010), comparing Kokei No. 14 and Beniharuka, which are widely used in Japan. The results are summarized in Supplemental Table 2. The plant, leaf, and storage root characteristics of Miyaakari are illustrated in Fig. 2. The growth habit of Miyaakari was ‘spreading’. Stem length and diameter of Miyaakari were longer and thinner, respectively, than those of Kokei No. 14 and Beniharuka. The pubescence of the tip in stems of Miyaakari was ‘medium’ and greater than those of Kokei No. 14 and Beniharuka. The anthocyanin coloration of nodes and internodes in Miyaakari was ‘absent or very weak’. The leaves of Miyaakari had a ‘cordate’ shape and were of ‘medium’ size. The color on both the upper and lower sides of the Miyaakari leaves was ‘light green’. The anthocyanin coloration on abaxial veins on the lower side of the Miyaakari leaves was ‘small’ and ‘weak’. The anthocyanin coloration of the nectary of the Miyaakari leaves was ‘absent or very weak’. Storage roots of Miyaakari had an ‘ovate’ shape, ‘red’ skin color, and ‘yellow’ flesh color. The depth of the eyes of the Miyaakari storage roots was ‘shallow’.

### Agronomic characteristics

The total yield of Miyaakari in the standard cultivation was 388 kg/a on average for 8 years, which was significantly higher than the total yields of Kokei No. 14 and Beniharuka for the same period (Table 1). The total yield of Miyaakari in the early harvesting cultivation was 166 kg/a on average for 3 years, which was significantly higher than that of Kokei No. 14 and approximately the same as that of Beniharuka (Table 2). The average sizes of the Miyaakari storage roots in the standard cultivation and the early harvesting cultivation were 196 g and 118 g, respectively (Tables 1, 2). The storage root size of Miyaakari was equal to that of Beniharuka and significantly larger than that of Kokei No. 14 in standard cultivation. The storage root size in the early harvesting cultivation was scarcely different among the cultivars. The number of storage roots per hill of Miyaakari in standard cultivation was 5.4, which is significantly higher than those of Kokei No. 14 and Beniharuka (Table 1). The number of Miyaakari storage roots per hill in the early harvesting cultivation was 3.8, which is also significantly higher than that of Kokei No. 14, with no significant difference from Beniharuka (Table 2). The dry matter contents of Miyaakari in standard cultivation and the early harvesting cultivation were 34.8 g/100 g FW and 30.5 g/100 g FW, respectively (Tables 1, 2). The dry matter content of Miyaakari was significantly higher than that of Kokei No. 14 and lower than that of Beniharuka in the standard cultivation, while there was no significant difference between Miyaakari and Kokei No. 14 in the early harvesting cultivation.

The resistance of Miyaakari and Kokei No. 14 to root-knot nematode, root lesion nematode and foot rot was evaluated in infested fields. The resistance to root-knot nematode was high in Miyaakari and low in Kokei No. 14; the resistance to root lesion nematode was moderately low in Miyaakari and intermediate in Kokei No. 14; the resistance to foot rot was moderately low in Miyaakari and Kokei No. 14.

### Quality of processed sweetpotato

The suitability of Miyaakari for three types of processed products, i.e., steamed sweetpotato paste, baked sweetpotato paste and diced boiled sweetpotato, were ranked as five points: poor = 1, good = 5) compared to Kokei No. 14 as the medium rank, 3 points. In the pastes of both the steamed and baked sweetpotato, Miyaakari was rated superior to Kokei No. 14 in all evaluated parameters, color, flavor, ease of straining, and overall evaluation (Supplemental Tables 3, 4). In the diced boiled sweetpotato, Miyaakari was rated as superior to Kokei No. 14 in color, flavor, and overall evaluation (Supplemental Table 5).

### Characterization of flesh colors

**Fig. 3.**
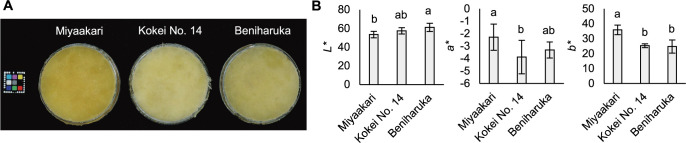
Characterization of the flesh color in Miyaakari, Kokei No. 14, and Beniharuka. Appearance (A) and L*a*b* values (B) of paste made of steamed sweetpotato.

**Fig. 4.**
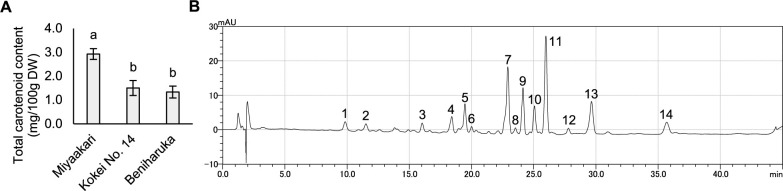
The carotenoid content in Miyaakari, Kokei No. 14, and Beniharuka and carotenoid composition in Miyaakari. Total carotenoid content in raw storage roots (A). Values followed by the same letter are not significantly different according to Tukey’s HSD test (*p* > 0.05). Chromatograms of carotenoids extracted from raw storage roots of Miyaakari (B). The peak identifications are 1: unknown, 2: unknown, 3: ipomoeaxanthin A, 4: unknown, 5: unknown, 6: ipomoeaxanthin C2, 7: β-crypthoxanthin 5,8-epoxide, 8: unknown, 9: β-carotene 5,8;5,8-diepoxide (cis-isomer), 10, 11: β-carotene 5,8;5,8-diepoxide (diastereomer), 12: unknown, 13: β-carotene 5,8-epoxide, 14: β-carotene.

Fig. 3A depicts the color of the pastes of steamed sweetpotato of Miyaakari, Kokei No. 14, and Beniharuka. In the paste of steamed sweetpotato, the L* value [indicating white(+)/black(–)] of Miyaakari was slightly lower than that of Beniharuka, and the a* values [indicating magenta(+)/green(–)] of Miyaakari was slightly higher than that of Kokei No. 14, while the b* value [indicating yellow(+)/blue(–)] of Miyaakari was significantly higher than those of both Kokei No. 14 and Beniharuka (Fig. 3B). The total carotenoid content of the raw storage roots of Miyaakari was significantly higher than those of Kokei No. 14 and Beniharuka (Fig. 4A). The carotenoids of Miyaakari were mainly 14 components (Fig. 4B). The carotenoids providing the highest values in Miyaakari storage roots was β-carotene 5,8;5,8-diepoxide (diastereomer), followed by β-crypthoxanthin 5,8-epoxide, β-carotene 5,8;5,8-diepoxide (cis-isomer), and β-carotene 5,8-epoxide. The main carotenoids in Kokei No. 14 and Beniharuka were also β-carotene 5,8;5,8-diepoxide (diastereomer), β-crypthoxanthin 5,8-epoxide and β-carotene 5,8;5,8-diepoxide (cis-isomer) (Supplemental Fig. 1).

### Change in the quality of storage roots during storage

Among the soluble sugars contained in steamed sweetpotato, two soluble sugars (glucose and sucrose) of Miyaakari and three soluble sugars (fructose, glucose, and sucrose) of Kokei No. 14 and Beniharuka were significantly increased by storing the storage roots for 12 weeks (Table 3). The total soluble sugar contents of Miyaakari and Kokei No. 14 were not altered by 12-week storage, whereas that of Beniharuka was significantly increased by 12 weeks storage. The total soluble sugar content of Miyaakari was significantly higher than that of Kokei No. 14 at 1 and 12 weeks of storage, while that of Miyaakari was at the same level as Beniharuka at 1-week storage and significantly lower than that of Beniharuka at 12 weeks’ storage.

The sweetness index in all cultivars was significantly increased by 12 weeks of storage, but the increases in the sweetness index of Miyaakari (1.38) and Kokei No. 14 (1.21) were relatively low compared to that of Beniharuka (2.67). The sweetness index of Miyaakari was at the same level as those of Kokei No. 14 and Beniharuka at 1 week of storage, while that of Miyaakari was significantly higher than that of Kokei No. 14 and lower than that of Beniharuka at 12 weeks’ storage.

**Fig. 5.**
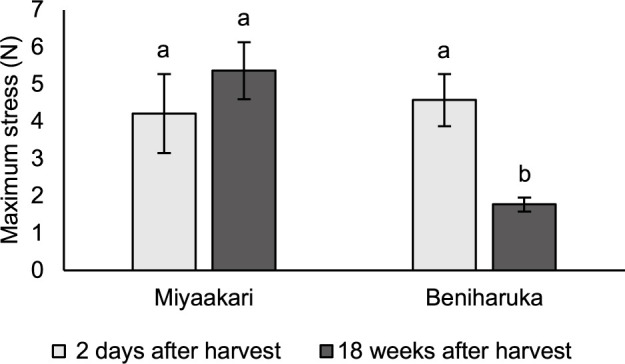
The hardness of steamed sweetpotato in Miyaakari and Beniharuka before and after storage. Values followed by the same letter are not significantly different according to Tukey’s HSD test (*p* > 0.05).

The hardness of the steamed sweetpotatoes was evaluated based on the maximum stress at cutting. The maximum stress for Miyaakari was not significantly altered between before and after storage, whereas that for Beniharuka was significantly decreased by 18 weeks of storage (Fig. 5). These results indicated that the hardness of steamed sweetpotato was changed by storage in Beniharuka and not changed by storage in Miyaakari.

## Discussion

Miyaakari has both high productivity and suitability for confectionery processing. Our present findings demonstrated that the yield of Miyaakari in standard cultivation was significantly higher than those of Kokei No. 14 and Beniharuka (Table 1). A high yield is required for sweetpotato cultivars to be used for food processing compared to cultivars for table use. Kokei No. 14 has been used for confectionery processing mainly in western Japan, but its yield is lower than those of recently released cultivars such as Beniharuka (Kai *et al.* 2010, Katayama *et al.* 2017). The cultivation of Miyaakari in place of Kokei No. 14 would therefore enable a more stable production of ingredients for confectioneries. We also observed that the yield of Miyaakari in the early harvesting cultivation was significantly higher than that of Kokei No. 14 (Table 2), indicating that Miyaakari is more suitable for early harvesting cultivation compared to Kokei No. 14.

Sweetpotato cultivars for food processing generally require a long period of cultivation because the yield obtained by early harvesting cultivation is lower than that obtained by standard cultivation. On the other hand, there are some merits of early harvesting cultivation; for example, the harvest can be sold at a relatively high price in summer when there are not many sweetpotato products on the market (MAFF 2024) and damage due to diseases and pests can be reduced (NARO 2023). Farmers are struggling with decrease of yield due to the spread of foot rot disease, and early harvesting cultivation, which shows fewer symptoms, is recommended. Miyaakari can achieve a reasonable yield and profit even with early harvesting cultivation. The early harvest cultivation of Miyaakari would provide these merits to farmers.

The suitability of the three sweetpotato cultivars for confectionery processing were evaluated by a primary food manufacturer, Agriprocess Miyazaki Company, Limited, which rated Miyaakari as superior to Kokei No. 14 for the application to all of the tested processed foods, paste of steamed and baked sweetpotato, and diced boiled sweetpotato (Supplemental Tables 3–5). The paste is widely used for ingredients of Japanese-style sweets (e.g., *imo-an* and *imo-yokan*) and western-style sweets (e.g., cream). The diced form is used for the topping of breads and cakes. The use of paste and diced Miyaakari should improve the quality of these sweets.

The high suitability of Miyaakari sweetpotato for confectionery processing is due to its flesh color. Confectionery manufacturers using sweetpotato paste tend to prefer paste with a deeper yellow color (Takano and Ino 2012). Our present study’s evaluation of b* values indicated that the yellow color of paste made of Miyaakari was stronger than those of Kokei No. 14 and Beniharuka (Fig. 3B), and the storage roots of Miyaakari included about twice the content of carotenoids compared to Kokei No. 14 and Beniharuka (Fig. 4A). Carotenoids are natural pigments showing yellow–red tones (Sun *et al.* 2022). The high carotenoid content resulted in the strong yellow present in the flesh color of Miyaakari.

Ishiguro *et al.* (2010) showed that the sweetpotato cultivar ‘Tamaotome’ contained the highest carotenoid content among eight yellow-fleshed varieties showing diverse flesh color from cream to yellow and about twice the carotenoid of Kokei No. 14. In terms of carotenoid content relative to that of Kokei No. 14, Miyaakari’s carotenoid content is comparable to that of Tamaotome. To further characterize the carotenoids of Miyaakari, we identified their components by HPLC, which revealed that the ratios of β-carotene 5,8;5,8-diepoxide and β-crypthoxanthin 5,8-epoxide in carotenoids of Miyaakari were high (Fig. 4B). These are main components in carotenoids of yellow-fleshed cultivars (Supplemental Fig. 1, Ishiguro *et al.* 2010). Our results suggested that the strong yellow color of Miyaakari is not caused by a certain carotenoid but rather by the total amount of each carotenoid.

In addition to flesh color, characteristics related to storage also indicate that Miyaakari is suitable for use in processed foods. The content and texture of sugars in heat-cooked sweetpotato are changed by post-harvest storage (Ando *et al.* 2018), and these changes affect physical properties of confectioneries for which the paste or diced form is used. An important problem is that the softening of sweetpotato during storage reduces the shape retention of paste and leads to deformed confectioneries (Takano and Ino 2012). The texture of Beniharuka is susceptible to softening by storage (Ando *et al.* 2018). As in earlier studies, the hardness of steamed sweetpotato was significantly decreased in Beniharuka in the present study (Fig. 5), and this changeable texture of Beniharuka during the storage period restricts its use for confectionery processing although the yield level of Beniharuka is higher than that of Kokei No. 14. Notably, Miyaakari can be used for confectionery processing in the same way regardless of the storage period because the hardness of steamed Miyaakari sweetpotato was not changed from before to after storage (Fig. 5). The sweetness index of Miyaakari was slightly increased by storage and was significantly higher than Kokei No. 14 after 12 weeks of storage (Table 3). Although most confectioneries do not require sweetness of the ingredients because the sweetness can be adjusted by adding sugar, some confectionery manufacturers prefer sweeter ingredients to reflect the original taste of ingredients in confectioneries (Takano and Ino 2012). Miyaakari may also meet this manufacturer preference.

The superior traits of Miyaakari would contribute to future breeding of sweetpotato. Miyaakari is suitable not only as an ingredient for confectioneries but also for consumption as a baked or steamed sweetpotato. Its bright flesh color is visually appetizing. However, the skin color of its storage roots is red, which is paler than that of Beniharuka. A purple red skin color, such as Beniharuka, is preferred on the fresh market in Japan. Improving the skin color of Miyaakari in breeding programs could lead to development of versatile cultivars for both processing and table use. Furthermore, Miyaakari shows higher resistance to root-knot nematode than Kokei No. 14. However, Miyaakari is moderately susceptible to foot rot, a susceptibility level equivalent to that of Kokei No. 14. Therefore, to ensure more stable production, a new cultivar for confectionery processing with resistance to multiple pests and diseases, including foot rot, will need to be developed.

## Author Contribution Statement

KS, YK, AK, TS, YK, TS, YT, MY, KK, and TF contributed to the breeding of Miyaakari. RK and KS analyzed the carotenoid and sugar components and investigated the characteristics during/after storage. KS drafted the manuscript, and all authors approved the final manuscript.

## Supplementary Material

Supplemental Figure

Supplemental Tables

## Figures and Tables

**Table 1.** The comparative performance of Miyaakari, Kokei No. 14, and Beniharuka in the standard cultivation

Parameter	Cultivar	Year								Mean*^a^*	*F-*value*^b^*		
		2015	2016	2017	2018	2019	2020	2021	2022		Year	Cultivar	Year × Cultivar
Total yield, kg/a	Miyaakari	348	395	353	373	416	417	426	379	388 a	8.57***	96.76***	2.11*
	Kokei No. 14	220	172	248	272	323	286	270	155	243 c			
	Beniharuka	299	333	349	320	342	397	398	290	341 b			
Avg. size of a storage root, g	Miyaakari	164	219	180	189	181	220	222	191	196 a	5.67***	24.46***	1.29
	Kokei No. 14	141	146	141	175	153	177	159	134	153 b			
	Beniharuka	157	208	179	223	159	226	202	216	196 a			
No. of storage roots per hill	Miyaakari	5.6	4.8	5.2	5.2	6.1	5.0	5.1	5.9	5.4 a	6.60***	20.38***	2.02*
	Kokei No. 14	4.1	3.1	4.6	4.1	5.5	4.3	4.6	3.3	4.2 c			
	Beniharuka	5.0	4.3	5.2	3.8	5.6	4.6	5.3	4.1	4.7 b			
Dry matter, g/100 g FW	Miyaakari	33.7	35.0	34.8	36.1	35.4	35.8	33.6	34.2	34.8 b	7.52***	201.89***	3.18**
	Kokei No. 14	31.9	31.5	31.3	34.3	33.5	33.3	31.9	30.5	32.3 c			
	Beniharuka	36.0	37.4	38.0	37.8	37.8	35.7	37.0	37.3	37.1 a			

*^a^* Values followed by the same letter are not significantly different in each trait according to Tukey’s HSD test (*p* > 0.05). *^b^* * *p* < 0.05, ** *p* < 0.01, *** *p* < 0.001 by two-way ANOVA.

**Table 2.** The comparative performance of Miyaakari, Kokei No. 14, and Beniharuka in early harvesting cultivation

Parameter	Cultivar	Year			Mean*^a^*	*F*-value*^b^*		
		2015	2016	2017		Year	Cultivar	Year × Cultivar
Total yield, kg/a	Miyaakari	151	151	196	166 a	39.14***	12.96*	2.01
	Kokei No. 14	96	101	187	128 b			
	Beniharuka	142	142	192	159 a			
Avg. size of a storage root, g	Miyaakari	104	116	133	118 a	25.78***	4.08	6.50**
	Kokei No. 14	111	98	139	116 a			
	Beniharuka	130	116	130	125 a			
No. of storage roots per hill	Miyaakari	3.8	3.6	3.9	3.8 a	16.21**	12.78**	2.56
	Kokei No. 14	2.3	2.7	3.5	2.8 a			
	Beniharuka	2.9	3.3	3.9	3.4 a			
Dry matter, g/100 g FW	Miyaakari	29.4	30.2	31.9	30.5 b	24.35***	8.18**	2.71
	Kokei No. 14	30.9	28.9	32.1	30.6 b			
	Beniharuka	32.7	33.7	34	33.5 a			

*^a^* Values followed by the same letter are not significantly different in each trait according to Tukey’s HSD test (*p* > 0.05). *^b^* * *p* < 0.05, ** *p* < 0.01, *** *p* < 0.001 by two-way ANOVA.

**Table 3.** The water content, soluble sugar content, and sweetness index of steamed sweetpotato of Miyaakari, Kokei No. 14, and Beniharuka at 1 and 12 weeks of storage

Cultivar	Storage period, wks	Water content, g/100 g FW*^a^*	Soluble sugar content, g/100 g FW*^a^*					Sweetness index*^a^*
			Fructose	Glucose	Sucrose	Maltose	Total	
Miyaakari	1	64.90 b	0.34 b	0.38 c	1.67 e	11.53 ab	13.91 b	6.25 cd
	12	63.87 b	0.46 b	0.63 b	3.06 b	10.74 b	14.89 b	7.63 b
Kokei No. 14	1	70.24 a	0.46 b	0.54 bc	1.77 de	8.55 c	11.32 c	5.52 d
	12	68.77 a	0.76 a	0.99 a	2.66 bc	7.89 c	12.31 c	6.73 c
Beniharuka	1	62.96 b	0.08 c	0.10 d	2.06 cd	11.94 a	14.19 b	6.38 c
	12	63.25 b	0.31 b	0.41 bc	4.24 a	12.22 a	17.17 a	9.04 a

*^a^* Values followed by the same letter are not significantly different among cultivars and storage periods in each soluble sugar content according to Tukey’s HSD test (*p* > 0.05, *n* = 3).
