# Supplementary material for: Breeding and characterization of a new sweetpotato cultivar, ‘Miyaakari’, with bright yellow flesh for confectionery processing
Source: Breed Sci. 2025 Oct 18;75(5):463–9. doi: 10.1270/jsbbs.25017 (PMC13129569; doi:10.1270/jsbbs.25017)
Supplement: Supplementary file 1 — Supplemental Figure [file 75_463_s1.pdf]

doi: 10.1270/jsbbs.25017

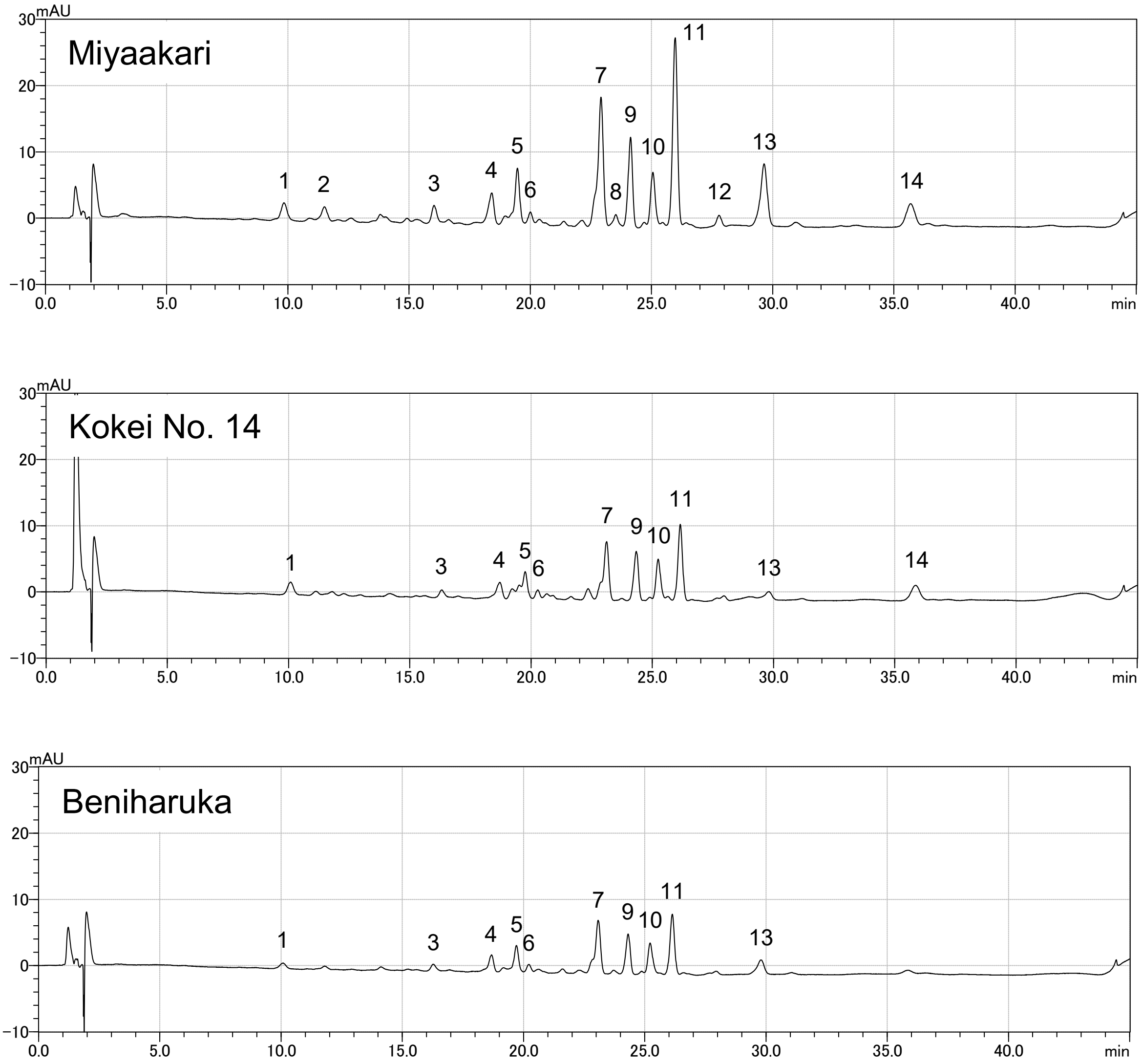

Supplemental Fig. 1. Comparison of the carotenoid compositions among Miyaakari, Kokei No. 14 and Beniharuka. The peak identifications are 1: unknown, 2: unknown, 3: ipomoeaxanthin A, 4: unknown, 5: unknown, 6: ipomoeaxanthin C2, 7: β-crypthoxanthin 5,8-epoxide, 8: unknown, 9: β-carotene 5,8;5,8-diepoxide (cis-isomer), 10, 11: β-carotene 5,8;5,8-diepoxide (diastereomer), 12: unknown, 13: β-carotene 5,8-epoxide, 14: β-carotene.
